# The use of postal audit and feedback among Irish General Practitioners for the self – management of antimicrobial prescribing: a qualitative study

**DOI:** 10.1186/s12875-022-01695-x

**Published:** 2022-04-18

**Authors:** Kevin F. Roche, Eimear C. Morrissey, Julie Cunningham, Gerard J. Molloy

**Affiliations:** 1 School of Psychology, National University of Ireland, Galway, Ireland; 2 Health Behaviour Change Research Group, School of Psychology, National University of Ireland, Galway, Ireland; 3 Continuing Medical Education Network, Irish College of General Practitioners, Dublin, Ireland

**Keywords:** Audit and feedback, Antibiotic prescribing, General practice, Qualitative research

## Abstract

**Objective:**

Inappropriate use of antibiotics has been acknowledged as a significant contributor to the proliferation of antimicrobial resistance worldwide. Physician prescribing of antibiotics has been identified as a factor in the inappropriate use of antibiotics. One methodology that is used in an attempt to alter physician prescribing behaviours is audit and feedback. This study aimed to explore the perceptions of Irish General Practitioners (GPs) towards the national introduction of postal feedback on their antibiotic prescribing behaviours beginning in 2019.

**Design:**

A qualitative descriptive methodology was used. Semi–structured interviews were conducted with GPs in receipt of postal audit and feedback.

**Method:**

GPs working in Ireland and in receipt of postal audit and feedback on their antibiotic prescribing behaviours participated in phone-based interviews. The interviews were recorded and transcribed verbatim. The collected data was then analysed using an inductive thematic analysis.

**Results:**

Twelve GPs participated in the study (female = 5). Three themes were identified from the analysis. The themes identified were *the reliability and validity of the feedback received*, *feedback on antibiotic prescribing is useful but limited* and *feedback needs to be easily digestible*.

**Conclusion:**

While the postal audit and feedback were broadly welcomed by the participants, the themes identified a perceived limitation in the quality of the feedback data, the perception of a likely low public health impact of the feedback and difficulties with efficiently processing the audit and feedback information. These findings can help refine future audit and feedback interventions on antibiotic prescribing.

**Supplementary Information:**

The online version contains supplementary material available at 10.1186/s12875-022-01695-x.

## Statement of contribution

### What is already known about this subject?

Antimicrobial resistance is an increasing issue for public health. The antibiotic prescribing behaviours of physicians is considered a key pathway for inappropriate antibiotic use. Previous research has identified that altering the antibiotic prescribing behaviour of physicians can have a positive influence on both the inappropriate use of antibiotics and the development of antimicrobial resistance. Audit and feedback has been used with varied success to alter the antibiotic prescribing behaviour of General Practitioners. In 2019 the Health Service Executive in Ireland began providing feedback to GPs on their antibiotic prescribing behaviour.

### What does this study add?

- This study garnered the perspectives of Irish based GPs towards the feedback they received.
- This study highlighted the barriers, both intrinsic and extrinsic, as to why such feedback is insufficient to improve antibiotic prescribing behaviours.
- This study highlights the need for, and benefit of GPs being involved in and having a greater understanding of the feedback design.

## Introduction

Antimicrobial resistance (AMR) is widely regarded as one of the most significant risks to global public health [1] with the overuse of antibiotics being identified as a key component of the advance of AMR [2]. A widely promoted solution to address the inappropriate prescribing and consumption of antibiotics at multiple levels is that of antibiotic stewardship [3].

Antibiotic stewardship has been described as a collective and multidisciplinary approach to improve the prescribing of antibiotics to improve clinical outcomes and to minimise the negative consequences of antibiotic use such as AMR [4]. While there has been an increase of hospital-based infections related to antimicrobial resistance [5], primary care settings are responsible for the majority of antibiotic prescriptions [3]. The Irish College of General Practitioners (ICGP) have defined the role of GPs in antibiotic stewardship stating that each GP should “…. prescribe the right antibiotic for the right patient at the right time with the right dose duration and route causing the least amount of harm to the patient and future patients” [6]. However, knowledge of the procedures and operation of antibiotic stewardship programmes in primary care settings, including their efficacy, remains unclear [7]. Further research in this area is important as previous research has found that GPs can be equivocal regarding the link between their own prescribing of antibiotics and the development of AMR [8].

Improving the antibiotic prescribing behaviours of physicians is a key facet of antibiotic stewardship and one methodology that is often employed for addressing this is the provision of audit and feedback, either on its own or as part of a multifaceted programmes, [9] and the provision of peer comparison audit and feedback has been shown to reduce antibiotic prescribing [10, 11]. A 2012 Cochrane review found that the use of audit and feedback on professional practices and healthcare outcomes had effects ranging from little or no effect to a substantial effect while rating the quality of evidence as moderate. It was also uncertain if the audit and feedback was more effective when combined with other interventions [12]. Factors that may have influenced the success of these interventions are that the audit and feedback component was part of a larger multifaceted programme and the participating prescribing physicians were high prescribers of antibiotics. A meta–ethnography in 2011 [13] listed seven factors that influenced a physician’s decision to prescribe antibiotics. One of the factors identified was external pressure to reduce prescribing and audit and feedback would appear to work through this route. However, in order for audit and feedback to be effective in reducing antibiotic prescribing rates it has been found that the disparity between the reported behaviour (the feedback) and the expected or desired behaviour must be quite large and the feedback must also be considered actionable [14]. There are numerous other factors that are likely to influence the efficacy of feedback in altering prescribing behaviour among primary care physicians, such as the source of the feedback, the perceived accuracy of the feedback and the design of the feedback [15].

### Current study

In September 2019 the Health Service Executive (HSE) in Ireland began disseminating feedback to GPs with patients on the General Medical Services (GMS) scheme. The GMS scheme provides free medical care and medications at point of access for people who are below the age of 6 years or aged 70 and above. In addition, patients who through either economic or health circumstances, would experience hardship in accessing healthcare are also eligible for the scheme. The scheme therefore over represents socio – economically deprived populations [16]. In 2018 43% of the Irish population were covered under this scheme [17]. The Antimicrobial Prescribing Report is issued by the Antimicrobial Resistance and Infection Control (AMRIC) Team in the HSE on a quarterly basis (https://www.hse.ie/eng/services/list/2/gp/antibiotic-prescribing/). The AMRIC team developed and published a preferred antibiotics initiative which issues guidance to GPs recommending the use of preferred narrow spectrum antibiotics (referred to as “green”) and avoidance of broad-spectrum antibiotics (referred to as “red”). The AMRIC team collaborated with the HSE Primary Care Reimbursement Service (PCRS) (The Primary Care Reimbursement Service is part of the HSE, and is responsible for making payments to healthcare professionals, like GPs, dentists and pharmacists, for the free or reduced costs services they provide to the public), to publish a quarterly feedback report for each GP in Ireland with a GMS panel >100 patients based on data derived through audit of antibiotic prescribing for all GMS patients. This commenced in September 2019. Each report details the prescribing of preferred and non-preferred antibiotics for patients on the GPs GMS panel and compares them with prescribing for all GMS patients within their Community Healthcare Organisation (CHO) area, and all GMS patients nationally. The data is presented in both actual prescription numbers, and percentage format. It places the individual GP percentage within a national quartile of low, mid-range or high for prescribing rates of preferred antibiotics. It also uses pie charts and tables to present the data, providing a breakdown by age group in the tables. One page of the report contains four tables, each one detailing the prescribing rates for one of four specific non-preferred antibiotics for each quartile of the previous twelve months. It provides an age group breakdown and compares the individual GP data with the national prescribing data.

This feedback was based predominantly on the typical antibiotic prescribing practices of the individual GPs for their patients on this scheme, however it was acknowledged that in some cases antibiotics could be prescribed by another prescriber e.g., out of hours’ service, another prescriber in the same practice or the prescription could originate in secondary care. The purpose of the current study is to evaluate and describe the perspectives of Irish GPs on the provision of postal audit and feedback for their antibiotic prescribing behaviours for their GMS patients.

## Method

The reporting of this study was conducted following the COnsolidated criteria for REporting Qualitative research (COREQ) reporting standards [18] and received ethical approval from the Research Ethics Committee of the School of Psychology at the National University of Ireland, Galway (NUIG) in January 2020 (Additional file 1).

### Methodology

A qualitative description approach as described by Sandelowski (2000) was used to explore the perceptions and experiences of GPs who had received audit and feedback. Qualitative description is particularly suitable for this work as it allows for an unadorned or ‘data-near’ description of the experiences of the participants [19].

### Interview development and piloting

The interview schedule development followed an iterative process beginning with general discussions among the research team of antimicrobial stewardship in primary care. These discussions identified the recent implementation of prescribing feedback to GPs on antibiotic prescribing following an automated prescribing audit. The research team then agreed that as the provision of the feedback was at an early phase that we would focus on the perspectives of GPs on this feedback, both the receiving the feedback and the format of the feedback. The relevant literature was consulted with a particular focus on the methods used in qualitative studies of prescribing papers. Further discussions among the research team about this prior literature resulted in the design of an interview schedule of 10 questions that would provide data suitable for the research objective. In early March 2020 a pilot interview was conducted in a face-to-face setting to ascertain the suitability of the interview protocol and interview guide framework in addressing the research question. After a review of the pilot interview transcript, it was felt that the interview schedule (Additional file 2) was suitable for the research objective.

### Recruitment of participants and impact of COVID-19

The targeted population of interest were GPs in Ireland working at least 2.5 days per week with a GMS list.

At the onset of the COVID-19 pandemic in Ireland a pilot interview had been conducted however a decision was taken to pause recruitment at this stage. Towards the end of April 2020 and after careful consideration of the dynamics of the COVID-19 pandemic situation in Ireland the research team felt that with modifications participant recruitment could resume. These modifications were that all interviews would be conducted remotely and the insertion of a question in the interview protocol regarding the impact of COVID −19 on the antibiotic prescribing behaviour of the participants.

Due to the nature of the situation at this time an adapted recruitment strategy was initiated where recruitment was conducted utilising a Continuing Medical Education Small Group Learning (CME-SGL) tutor of the Irish College of General Practitioners (ICGP) (for a brief overview of the CME-SGL see [20]). Using an opportunistic sampling strategy, the CME-SGL tutor identified potential participants, who were regular attenders of CME – SGL, who they felt would be willing to participate in the research and made the initial contact with them. All those contacted expressed interest in participating in the research then they were forwarded a document packet electronically comprising of an invitation letter to participate in the research, a copy of the consent form which could be signed electronically and returned or printed and a signed physical copy returned, the participant information sheet and an anonymised example copy of the audit and feedback summary that the GPs receive on their antibiotic prescribing. GPs who then confirmed willingness to participate were contacted to arrange a suitable time for the interview to be conducted. Thirteen GPs from the midlands area of Ireland agreed to participate with ten located in urban settings and three in rural settings, (where a rural setting is considered to have a population density of less than 1500). There were no incentives offered for participation in the research. At the time the interviews were conducted (May – June 2020) the participants had received at least three antibiotic prescribing reports. While the number of participants in the present study (*n* = 12) has been found previously to be sufficient for data saturation [21] it should be noted that the concept of data saturation within reflexive thematic analysis has been described as neither useful nor theoretically coherent [22].

### Data collection

There were a total of 13 semi – structured interviews conducted, one pilot interview and twelve research interviews. Prior to undertaking further data collection, the protocol was altered as discussed above considering the COVID −19 pandemic and all interviews were conducted remotely. All the interviews were conducted by one researcher (KR), a male trainee health psychologist (BA, MSc) with experience in interviewing and qualitative research. All interviews were audio recorded with the permission of each participant. Nine of the interviews were conducted during office hours, with four (including the pilot) conducted after 6 p.m. The interviews ranged in duration from 8 to 26 min. Two of the participants were known informally to the interviewer.

To facilitate the interviews in as timely a manner as possible, only the questions in the interview guide were asked allowing the data to be collected with minimal influence from the researcher’s beliefs or opinions or previous interview data. Prior to the interviews each participant was provided with an anonymised version of the feedback sheets electronically and asked to have them or their own feedback available during the interview so that they could reflect on the design and layout of the feedback.

After completion of the first two interviews another member of the research team (GM) reviewed the transcripts and gave feedback on the collected data, and it was agreed to proceed without altering the interview protocol or interview guide. A preliminary analysis was conducted throughout the data collection phase and no new themes were identified after the tenth interview.

### Data analysis

Interview transcription was carried out by the research team with all the interviews being transcribed verbatim. The transcripts were not returned to the participants for comment. Due to differences in the interview schedule and setting of the interview between the pilot and the remaining interviews the data from the pilot interview was not used in the analysis.

The generated data was analysed inductively which allowed for the identification of themes without the use of a previously developed theoretical framework or being overly influenced by the researcher’s perspective [23]. The initial analysis and theme identification was conducted by KR under supervision of GM. This involved regular meetings to discuss the process of the analyses, the participant responses and the identification of the recurrent themes. The themes identified were subsequently discussed and refined with a third member of the team (EM). The interview data was reviewed throughout the period of data collection and no themes/issues were identified that required refinement of interview protocol or guide. The data was coded, and the themes identified at a semantic level [23].

Following from the initial coding and generating of themes and sub – themes the research team met to discuss the identified themes. This involved the lead researcher (KR), the research supervisor (GM) and a third member (EM) in the role of “critical friend”. This meeting allowed the themes to be discussed and the language used to label the themes clarified and reified.

## Results

### Participants

There were a total of 13 GPs recruited. 1 GP (Female, 20+ years qualified) participated in the pilot interview session. Of the remaining 12 (Female = 5) GPs that participated in the research 2 were qualified in General Practice less than 10 years, 6 were qualified between 10 and 20 years prior to the research and 2 were over 20 years qualified as GPs. The participants were asked to estimate the percentage ratio of private and public (GMS) patients that they would see on an average day with the majority (6) estimating 50:50. 4 estimated that they would see 60:40 and 2 estimated 70:30 public to private. The generated themes and sub – themes are presented in Table 1.

**Table 1:** Themes and sub – themes generated from the data

*Themes*	*Sub – themes*
Reliability and validity of the feedback received	GPs have incomplete autonomy over prescribing including prescribing that impacts their feedback Confusion over how the feedback data is derived
Audit and feedback is useful but is of limited impact for antimicrobial prescribing	Increased awareness among GPs around antimicrobial prescribing behaviours Forces outside General Practice drive demand and use of antimicrobials
Format of the audit and feedback data that GPs receive	

### Theme 1: reliability and validity of feedback received

The theme of reliability and validity of the feedback that the GPs receive was emphasised by nearly all the participants. Many participants expressed confusion as to how the prescribing feedback data is derived and expressed doubts over its reliability as a result.

*do they refer to patients who are registered with me or patients that were done on my prescription pad?*

*(Participant 8)*

### Subtheme: autonomy over prescribing

Several of the participants referred to not having complete control over the prescribing that they were receiving the feedback for. A key issue seemed to be prescriptions that were generated from other sources were bring transcribed onto the GPs prescription pad, to enable the patient to access the lower medication costs that come with the GMS.

*a lot of times GPs will transcribe what maybe came from a hospital onto GMS paper or from the dentist onto GMS paper and so some of the antibiotics perhaps were you know more extensively used in your GMS reporting than what you may have actually primarily prescribed*

*(Participant 1)*

This subtheme is seen in several of the individual interviews with participants discussing either other doctors in their practices, or doctors in out of hours’ services prescribing non-preferred antibiotics for their GMS patients or transcribing prescriptions from hospitals resulting in feedback being given on prescribing that was not actually carried out by the GP.

*an awful lot of the time you’re just transcribing a prescription from a consultant*

*(Participant 5)*

*my partner prescribes to patients who are registered with me all the time* …. and *my partner prescribes Augmentin a lot more than I do*

*(Participant 8)*

### Subtheme: confusion over how the feedback data is derived

GPs appeared to have several different interpretations of how their feedback data is derived. This varied from an interpretation that the feedback is based solely on prescriptions written by the individual participants, to the interpretation that it is based on any prescribing to GMS patients registered to a particular participant (which is the correct interpretation).

*I’m kinda not sure about this but you know somebody writes a prescription for a patient of mine but it’s under a different GMS list I don’t actually know whether that’s a reflection or reflected on my prescribing or whether it goes on the list of the name of the person on whose pad it is*

*(Participant 10)*

This participant expressed a lack of clarity around whether feedback comes from the prescription pad or which GP the patient is registered to. There are various other examples of this type of confusion expressed throughout the interviews with only three participants (1, 2 and 5) voicing the same interpretation, assumed to be correct, as the research team. The general confusion around this issue was explicitly voiced by one participant:

*I’m still confused as to whether these are actually my true figures*

*(Participant 8)*

This confusion around the feedback data meant that some participants were unsure of whether the feedback they received was true to their own practice, and participants who were certain that the feedback was not reflective of their own antibiotic prescribing behaviour reported that the feedback had a limited impact it had on their practice.

***Interviewer:*** *Has receiving the feedback altered your prescribing habits do you think?*

***Participant:*** *Not particularly because I have already kind of altered my prescribing habits on national guidelines, rather than on the feedback from the audit*

***Interviewer:*** *Okay okay.*

***Participant:*** *Especially in view of the fact that I know it's not necessarily stuff that I prescribed, it could be coming from A&E discharges that are transcribed to GMS scripts or dental scripts that come in and they're the ones that use more of the non-preferred antibiotic, like Augmentin all the time we don't use that for much other than say abscesses or certain gastrointestinal problems*

### Theme 2: audit and feedback is useful but is of limited impact for antimicrobial prescribing

Many participants reported that they found the feedback useful because it focused their attention on their individual antibiotic prescribing behaviours and increased their awareness of the issues surrounding antibiotic prescribing and antimicrobial awareness. However, there was a recognition that impact was limited because of external factors. Accordingly, the two sub – themes generated from this theme were the *feedback resulting in an increased awareness of prescribing behaviours* and the *influence of outside forces on antibiotic prescribing.*

### Subtheme: increased awareness leading to a change in antibiotic prescribing behaviours

This sub – theme was identified in the data with the majority of participants acknowledging that the feedback led to an increased awareness of their own antibiotic prescribing behaviours and a belief that this type of feedback would help in changing the antibiotic prescribing behaviours of GPs in general. Participants specifically referred to prescribing preferred rather than non-preferred antibiotics as a result of receiving the feedback.

*I would notice myself instead of a macrolide I would be more inclined to prescribe doxycycline and tetracycline I possibly would be prescribing more Amoxicillin than Augmentin now*

*(Participant 5)*

*but yeah, it definitely made me focus more on what I should be doing rather than not doing*

*(Participant 3)*

Those participants who felt that they had not altered their prescribing behaviours attributed it to a prior high awareness of the issues surrounding antibiotic prescribing and antimicrobial resistance.

*I paid more attention to what was being recommended by the experts in microbiology*

*(Participant 9)*

### Subtheme: outside Influences

All participants expressed a range of outside influences as having a bearing on antibiotic use. Patient demand featured strongly throughout many of the interviews with a focus on demand for “stronger” antibiotics. The data suggests that even though GPs recognise that prescribing a particular antibiotic may be inappropriate, they can feel pressured into prescribing it due to the nature of the GP / patient relationship or the time constraints of the consultation period. In the current study participants reported that some patients expressed preferences for non-generic antibiotics or what patients viewed as strong antibiotics.

*But you know this idea that erm “don't give me the generic” that's rife in Irish practice*

*(Participant 10)*

*you feel then maybe you’re prescribing inappropriately because of the patient’s expectation*

*(Participant 1)*

*patient pressure so like as in they have expectations that Augmentin works for them and it’s a strong one and that’s the one they need and you’re busy and you just don’t have time to spend that extra ten minutes in a consultation explaining to them there’s no such thing as strong antibiotics (Participant 4)*

The COVID – 19 pandemic was also considered by some of the participants as having a varied effect on antibiotic prescribing resulting in changes to prescribing behaviours, positively for some participants and negatively for others with some participants reporting that they felt less pressurised into prescribing antibiotics because of the move to remote consultations.

*so, if they’re not in front of you feel under less pressure, I think to prescribe something*

*(Participant 2)*

However, some participants reported the converse, where a remote consultation without physical exam meant that they were more inclined to prescribe antibiotics even if they were unsure if it was appropriate:

*Because I can’t examine them I can’t tell them that there isn’t pus in their throat and there isn’t glands up and temperature isn’t up so you’re going by what the story the parent tells you they’re just getting antibiotics (Participant 4)*

### Theme 3: format of audit and feedback

The format of the audit and feedback data sent to GPs was identified as a theme in the majority of the participant interviews. All participants liked the pie charts, with Participant 10 referring to them as “*pretty straightforward*”.

This visual mode of presenting data was much preferred to other text-heavy modes with one participant stating that they found the first sheet with the pie charts *“very user friendly”* and referring to the second sheet containing feedback tables as *“a little bit less user friendly” (Participant 1).* Other participants were more vociferous in their criticism of the text heavy second sheet

*actually, there’s a lot of data on it kinda hurts your eyes when you look at it*

*(Participant 3)*

A majority of the other participants echo this sentiment throughoutthe data with opinions expressed such as “*It’s too wordy and too complicated” (Participant 4)* and *“I’m not sure what I’m looking at half the time” (Participant 11).*

The time it takes to read and digest the feedback data is a factor.

*It’s quite a busy page takes a little while to understand what’s on it*

*(Participant 3)*

*I don’t have time for that*

*(Participant 4)*

*when you’re very busy it’s very difficult to sit down and study all these numbers and percentages*

*(Participant 12)*

Thus, participants described how the presentation of data visually was preferable when time available for interpretation of the feedback was pressured or limited.

## Discussion

### Summary

The present research examined the perspectives of Irish GPs on the use of a new postal audit and feedback for antimicrobial prescribing. The provision of the feedback was broadly welcomed, and participants reported an increased awareness of their prescribing behaviours subsequently. However, attention was drawn to some limitations with the feedback. There was confusion over whether the feedback data was derived from the individual prescriber, or whether all prescriptions were written for patients on the doctors’ GMS panel. This lack of clarity reduced the credibility of the data from the perspective of the participants. Certain aspects of the feedback design were also reported as being overly complex for processing in time pressured working environments with visual presentation of the data being preferable.

### Strengths and limitations of the research

There are methodological limitations to this study as it investigated a cohort in one region, as opposed to nationally, due to COVID-19 and therefore the findings may not be an accurate reflection on the perspectives of GPs nationally. The impact of the COVID-19 pandemic must also be highlighted as it led to altered work practices and increased stress among participants. At the time the research was conducted GPs had received either three or four, depending on when interviews were conducted, rounds of feedback. As GPs continue to receive feedback on their antibiotic prescribing behaviours and become increasingly familiar with the format of the feedback data the cognitive effort required to interpret the feedback may decrease.

A key strength of this research is that it was conducted at an early phase of the provision of the antibiotic prescribing feedback to GPs and therefore the research team were in a position to share the findings with the team responsible for designing the format of the audit and feedback and to provide design input to later iterations. The multidisciplinary composition of the research team (2 Health psychologists, 1 GP & 1 trainee Health Psychologist) was an additional methodological strength. Having a GP involved in the recruitment process resulted in 100% recruitment (13 GPs were approached and all agreed to participate) and also in reviewing sections of the transcripts where the participants used medical terminology or professional jargon to ensure the interpretation was correct in the analysis. The health psychology input ensured that there was appropriate behavioural specificity in the gathering, analysis and interpretation of the qualitative data on the antibiotic prescribing behaviour of GPs.

### Comparison of present research with existing literature

The use of audit and feedback for prescribing behaviours of antimicrobials in general practice has increased in prevalence in recent years with some success [11, 24, 25]. However, these studies are based in other countries operating with different health systems, being subject to different legislation and with different data repositories from which to base the feedback on. The audit and feedback may also be incorporated with other interventions [24] or focus specifically on one cohort or infection type [25]. In Ireland, data for the prescribing of antibiotics in primary care has until recently not been readily available [26]. The same research found that there is a concerning amount of prescribing of broad-spectrum antibiotics among adult populations in Ireland. It was also found that one particular first line antibiotic, Amoxicillin, was being utilised for a placebo effect as opposed to being used as a specific treatment for a diagnosed bacterial infection [26].

The use of feedback to inform GPs of their antibiotic prescribing behaviours is a systemic change to studies that have reported that Irish GPs rarely received any formal feedback on their prescribing behaviours [27]. In this study, the use of feedback was generally welcomed by the participants. A regional cluster randomized complex intervention to improve antimicrobial prescribing for urinary tract infection in Irish general practice was reported in 2016 [28]. A combination of workshop, information on antimicrobial prescribing guidelines, a practice audit report and a reminder integrated into the patient management software suggesting first-line treatments was developed. The proportion of antimicrobial prescribing according to guidelines for urinary tract infection increased significantly and this effect was sustained at 5 months after the intervention finished. However, this was delivered in one region only and for a limited period.

Three themes were generated from the collected data on the perspectives of the participants on the feedback that they received. The three themes address the original research objective of exploring the perspectives of Irish GPs towards the national introduction of postal feedback on their antibiotic prescribing behaviours and also identify other pertinent factors in the prescribing of antibiotics in general practice in Ireland. The reliability and validity of feedback has an effect on the credibility ascribed to the feedback data. Credibility has been previously identified as having an impact on the efficacy of the feedback [15, 29]. This was reflected in this study where participants who did not believe that the feedback reflected their prescribing practices also reporting that the feedback had a limited impact on their behaviour. The usefulness but limited impact of audit and feedback was previously described by [12], with baseline performance and how the feedback is provided having an effect on physician behaviours. There have been similar findings where audit and feedback was shown to have an effect on high prescribers of antibiotics but the prescribing of non – preferred antibiotics was attributed to other sources (out of hours facilities, secondary care and dental surgeries) [11] and so the efficacy of the feedback was both directly and indirectly (through the perceived credibility of the feedback) limited. The format of the audit and feedback was also identified by participants as being a factor. The format and design of feedback is an important aspect in the effectiveness of feedback [30] as poor feedback design will limit participant engagement.

These themes suggest that just sending out feedback on antibiotic prescribing behaviours to GPs is unlikely to be enough to bring about a large change on the amount of antibiotic use in primary care in Ireland in the short term. Outside of the current study it should be noted that previous research which has found audit and feedback to be successful has targeted GPs who were acknowledged high prescribers of antibiotics [11]. Evidence synthesis of audit and feedback has been shown to lead to small but potentially clinically important improvements in professional practice such as prescribing patterns. A 2012 study [31] has reported that the use of audit and feedback in an American paediatric hospital resulted in a 7% overall reduction in antimicrobial prescribing. The Cochrane review [12] referred to previously, found that the use of audit and feedback resulted in a median improvement in healthcare professionals desired behaviours of 4.3% over 140 randomised control trials. These figures may appear initially quite modest; however, when considered at a population health level the potential cumulative gains due to the iteration of the feedback has the potential to lead to substantive change and a meaningful improvement in public health.

There are numerous psychological factors that can influence the success of audit and feedback methodologies in altering behaviour. Relating to the themes identified in this study the *format of the feedback data that the GPs received* is of particular salience in this area as when the feedback presented is overly complicated in design it can cause an excessive cognitive load [15]. Cognitive load refers to the effort required to process i﻿nformation and good feedback design will limit the cognitive load experiences by the recipients [15]. While the data shows that participants approved of the graphical representation of the feedback there was a general consensus that the rest of the feedback was overly complicated and required too much time and effort to interpret.

The current study found that GPs have a good awareness of AMR and the role that antibiotic prescribing can play. None of the participants expressed any doubt that antibiotic prescribing in general practice is a contributing factor to AMR. This study highlighted the limitations of the current provision of the feedback. By expressing doubts as to the reliability and validity of the feedback data the participants give voice to findings of previous research that for feedback to be effective the recipients must perceive the feedback data as being credible [29]. This is also expressed in the two sub – themes where participants expressed that they do not have complete autonomy over the prescribing that impacts on their feedback and the confusion as to how the feedback data is derived. Attending to the credibility of the data utilised in the provision of the feedback is necessary for the feedback to be successful in eliciting a change in behaviour [15]. Considering the early stage of the feedback provision that this research was conducted future work in this area could apply the use of a framework, such as the Clinical Performance Intervention Theory (CP-FIT) framework [32] that has previously been used to assess a pilot quality improvement program designed to support appropriate antimicrobial prescribing in Australian General Practice [33].

### Implications

There are many possible future research directions from this study. Some of these could include the use of GPs in feedback design and the targeting of other influences on inappropriate prescribing, such as patient demand. Research into the antibiotic prescribing behaviours of hospital-based medics, as mentioned frequently by participants, should also be considered to provide insight into a different pathway for inappropriate antibiotic use. There is also potential to develop further interventions to enhance the use of audit and feedback to effect change in antibiotic prescribing behaviours. There are two main practical implications from this research specifically for the feedback. The first is that there are issues surrounding the veracity of the data used to generate the feedback, namely the reliability and validity and second that the GPs involved in this study felt that the presentation of the feedback could be improved in ways that allow it to more efficiently interpreted and digested e.g. through simplification of the data presentation. Another practical implication is that the participants expressed the belief that feedback by itself would not be sufficient to address inappropriate antibiotic prescribing. Practically some of these issues could be addressed by including GPs in the design of the later iterations of the feedback in order to make them more user friendly.

## Conclusion

The objective of this research was to gather the perspectives of Irish GPs on the provision of feedback on the prescribing of antibiotics to their public patients. The research found that while there is broad support for the feedback among the participants there are a number of perceived limitations in its current format. However, as the provision of this feedback is still at an early stage there is opportunity for improvements to be integrated. This study found support for previous research on the use of audit and feedback for antibiotic prescribing in Primary Care / General Practice and that behavioural science approaches can contribute to addressing the overarching issue of antimicrobial resistance in these settings.

## Supplementary Information

**Additional file 1.**

**Additional file 2.**

## Data Availability

For inquiries regarding data supporting the findings presented in the manuscript, please contact the corresponding author.

## Abbreviations

GPs: General Practitioners
AMR: Antimicrobial resistance
ICGP: Irish College of General Practitioners
HSE: Health Service Executive
GMS: General Medical Services
CME – SGL: Continuing Medical Education – Small Group Learning
AMRIC: Antimicrobial Resistance and Infection Control
PCRS: Primary Care Reimbursement Service
COREQ: Consolidated Criteria for Reporting Qualitative Research
