# Supplementary material for: The use of postal audit and feedback among Irish General Practitioners for the self – management of antimicrobial prescribing: a qualitative study
Source: BMC Prim Care. 2022 Apr 18;23:86. doi: 10.1186/s12875-022-01695-x (PMC9014781; doi:10.1186/s12875-022-01695-x)
Supplement: Supplementary file 2 — Additional file 2. [file 12875_2022_1695_MOESM2_ESM.docx]

**The use of postal audit and feedback among Irish General Practitioners for the self – management of antimicrobial prescribing: A qualitative study.**

**Interview schedule**

**Questions** (& additional prompts if required)

**1. Can you recall your reaction when you received the initial letter informing you that the HSE were beginning to send GMS list holders a report on their antibiotic prescribing for GMS patients?**

**2. When you received the first quarterly report, that is the feedback, what was your reaction?**(1) To receiving the feedback first off
(2) To the specific feedback for you it contained

**3. Do you feel that the feedback you received is an accurate reflection of your antibiotic prescribing behaviour in general? (if yes / no, then why?)**Do other doctors in the practice prescribe for patients on your GMS list?
Do you think antibiotic prescribing in out of hours services has an impact on the feedback you received?
What about following hospital consultant recommendations?

**4. Has receiving the feedback altered your prescribing habits do you think?**(if yes: “in what way?”) (if no: “why not?”)

**5. Looking at the layout of the feedback you received specifically pages 5 and 6 do you think it could be improved?**

**6. Do you think that GPs receiving feedback in this format will help change antibiotic prescribing patterns?**

**7. Do you feel that addressing antibiotic prescribing by GPs can have an effect on the issue of antimicrobial resistance?**

**8. Do you have any further comments about the feedback you received specifically?**

**9. Do you think that your antibiotic prescribing has changed since the onset of the COVID-19 pandemic?**

**10. Do you have any further comments about the feedback you received in general?**

**11. Do you have any further comments about antibiotic prescribing in General Practice?**
